# In-vitro and in-vivo assessment of the bactericidal potential of peracetic acid and hydrogen peroxide disinfectants against *A. hydrophila* infection in Nile tilapia and their effect on water quality indices and fish stress biomarkers

**DOI:** 10.1038/s41598-024-76036-2

**Published:** 2024-10-28

**Authors:** Abdelrhman Gamal, Dalia A. Abdel-moneam, Asmaa Safwat Morsi, Nermeen M. L. Malak, Asmaa Metwally Ali, Hanan S. Khalefa

**Affiliations:** 1 Department of Veterinary Hygiene and Management Faculty of Veterinary Medicine, Cairo University, Giza, 12211 Egypt; 2 Department of Aquatic Animal Medicine and Management, Faculty of Veterinary Medicine, Cairo University, Giza, 12211 Egypt; 3 Department of Physiology, Faculty of Veterinary Medicine, Cairo University, Giza, 12211 Egypt; 4 Department of Food Hygiene and Control, Faculty of Veterinary Medicine, Cairo University, Giza, 12211 Egypt

**Keywords:** Peracetic acid, Hydrogen peroxide, *A. hydrophila*, Bacterial count, Cortisol, Antioxidant capacity, Ecology, Microbiology, Molecular biology, Physiology, Biogeochemistry

## Abstract

This study aimed to assess the in vitro and in vivo disinfectant potential of peracetic acid (PAA) (1 mg/L) and hydrogen peroxide (H_2_O_2_) (20 mg/L) on the physicochemical and microbiological water quality parameters of fish aquaria, the microbial density of Nile tilapia muscular tissue, fish hepatic cortisol levels, and antioxidant biomarkers. In vitro, PAA and H_2_O_2_ reduced *A. hydrophila* colony viability by 5 log units after 30 and 5 min of contact time, respectively. PAA and H_2_O_2_ were added to aquaria water twice a week for the three-week experiment. Increased fish escape reflexes were observed only in the PAA group, which returned to normal within 10 min. No mortalities were reported in either the PAA or H_2_O_2_ groups. An in vivo experimental challenge with a pathogenic strain of *A. hydrophila* revealed a 20% reduction in mortality in the PAA group, with no mortalities in the H_2_O_2_ group. Cortisol levels and antioxidant markers were measured to assess the impact of PAA and H_2_O_2_ on fish health. Cortisol levels in the PAA and H_2_O_2_ groups were significantly higher than in the control group after disinfectant exposure, but they progressively returned to normal. A significant reduction in superoxide dismutase (SOD) and catalase (CAT) activity, along with considerably higher glutathione peroxidase (GPx) and malondialdehyde (MDA) enzymatic activity, was observed in the PAA and H_2_O_2_ groups compared to the control group. A substantial increase in total antioxidant capacity (TAC) was recorded in the PAA group. Physicochemical analyses revealed reduced pH and increased dissolved oxygen levels in the PAA and H_2_O_2_ groups. Microbiological analyses showed a significant reduction in bacterial density in water by 64% and 76% after 30 min of exposure to PAA and H_2_O_2_, respectively, with a non-significant increase in microbial count after bacterial challenge. Additionally, aerobic bacterial count, *Aeromonas spp*., and psychotropic bacterial count in fish muscle showed a significant reduction in the H_2_O_2_ group compared to the PAA and control groups before and after infection. The study concludes that regular application of PAA and H_2_O_2_ can temporarily reduce bacterial load in aquaria and fish muscle, regulate stress responses, and improve fish health by reducing *A. hydrophila*-induced infections and improving survival.

**Supplementary Information:**

The online version contains supplementary material available at 10.1038/s41598-024-76036-2.

## Introduction

Bacterial disease outbreaks pose a significant challenge to freshwater and mariculture systems, causing up to a 50% loss in *Oreochromis niloticus* fish production in Egypt^1–3^. In recent years, *Aeromonas* species have been the primary cause of mass mortality outbreaks in aquaculture during the summer, leading to hemorrhagic septicemia^4,5^. This opportunistic pathogen infects fish when a key factor disrupts the balance between pathogen, host, and environment. The heterogeneity of *Aeromonas* strains hinders vaccine development, making water disinfectants a viable alternative for preventing and controlling bacterial infections^6^.

Maintaining preventive measures is crucial for managing fish diseases. Several low-cost biosecurity approaches have proven effective and easy to implement in aquaculture, including systemic treatments with antibiotics, immunostimulants, feed additives, water disinfectants, or a combination of these measures^7,8^. Disinfectants and synthetic chemicals are widely used for disease prevention and control in fish and other aquatic species^9^. The disinfectant application frequency depends on the infection severity and water conditions^10^.

Peracetic acid (PAA) and hydrogen peroxide (H_2_O_2_) are environmentally friendly disinfectants that decompose into harmless by-products, releasing no pollutants^11^. These agents are effective against various aquatic pathogens, with their microbicidal activity depending on concentration and ratio. PAA is increasingly used in aquaculture as an antibacterial agent for therapeutic and disinfection purposes^12,13^. Unlike other disinfectants (formalin, chlorine, and ozone), PAA has a wide safety margin, lacks carcinogenic risks, produces negligible harmful by-products, and poses minimal health hazards^14^. It rapidly dissociates into acetic acid and hydrogen peroxide in the presence of microorganisms, leaving no residue^15^. PAA’s primary mechanism of action involves oxidation, leading to bacterial cell membrane rupture and inhibition of bacterial colonization^16^. Its efficacy in fish disease control has been demonstrated in several studies^14–17^.

Hydrogen peroxide is a potent oxidizing agent that kills bacteria by generating hydroxyl free radicals, attacking and destroying bacterial DNA, cell membranes, and other essential cellular components^18^. Additionally, H_2_O_2_ enhances water quality by increasing oxygen levels through its dissociation^19^. Factors such as water salinity, temperature, organic load, biofilter performance, fish feeding rate, and stocking density significantly influence the amount of H_2_O_2_ required. H_2_O_2_ is favored over other chemotherapeutic agents due to its rapid breakdown into harmless by-products in aquaculture systems^18^. However, excessive, uncontrolled disinfectant exposure can lead to oxidative stress and toxicity, necessitating careful management practices^20^. Conditioning fish prior to treatment can help mitigate stress^11^.

Nile tilapia is one of Egypt’s most popular cultivated fish species^21^. It is highly favored by consumers due to its affordability and high nutritional value, providing an excellent source of animal protein, rich in essential amino acids, critical polyunsaturated fatty acids, and omega-3s^22^. However, the fish’s exposure to microbial pathogens such as *Aeromonas* species during cultivation, harvesting, handling, and processing makes it highly perishable, posing potential health risks to humans^23^. Therefore, this study aimed to conduct a parallel and comparative experimental trial to evaluate the effects of short-term exposure to PAA and H_2_O_2_ as feasible disinfectants on the physicochemical and microbiological water quality in fish aquaria, microbial load in fish muscle, and fish stress responses, including cortisol levels, antioxidant biomarkers, and the protective effects of these disinfectants against *Aeromonas hydrophila* infection.

## Materials and methods

### Tested disinfectants

This study utilized two types of commercial disinfectants. EGY-Virox^®^ (Egy-Holland, Egypt) describes the per acetic acid disinfectant as contains 5% PAA and 25% H_2_O_2_, with a PAA: H_2_O_2_ ratio of 0.2, and a chemical stabilizer to prevent PAA degradation in the aqueous solution. The hydrogen peroxide disinfectant, AQUAPLUS^®^ (Egy-Holland, Egypt), comprises 50% stabilized H_2_O_2_ and organic acids.

### Fish bacterial identification

#### Isolation and phenotypic characterization

The pathogenic bacterial isolate used in this study was *Aeromonas hydrophila*, recovered from a previous tilapia mass mortality event. The infected fish’s surface was sterilized with 70% ethyl alcohol. Kidney samples were obtained, inoculated onto brain-heart infusion (BHI) broth (LabM, UK), and incubated overnight at 29 °C. Turbid broth samples were then streaked onto Aeromonas agar base media and Rimler-Shotts (R-S) media (Hi-Media, India). Purified *A. hydrophila* colonies were identified using conventional methods, including colonial morphology, gram staining, motility testing, oxidase tests, catalase tests, and analytical profile indexing (API 20E) kits (Biomerieux, France). For further molecular investigations, pure isolates were stored in BHI with 15% glycerol at − 20 °C (v/v).

#### Molecular identification and sequencing of isolated bacteria

*A. hydrophila* DNA was extracted using the Quick-DNATM Fungal/Bacterial Miniprep Kit (Zymo Research Products, USA), then purified with DNA Clean and Concentrator^®^-25 (Zymo Research Products, USA). The PCR reaction was conducted in 50 µL aliquots using COSMO PCR RED Master Mix (Willowfort, Birmingham, UK), with universal 16 S rRNA gene amplification (F: 5′-AGAGTTTGATCCTGGCTCAG-3′ and R: 5′-GGTTACCTTGTTACGACTT-3′) following Weisburg et al.^24^. The purified DNA was sequenced using the ABI 3730xl DNA sequencer (Applied Biosystems TM). The obtained sequence was blasted against the National Center for Biotechnology Information (NCBI) database and registered in GenBank to obtain an accession number.

#### Phylogenetic analysis

The obtained *A. hydrophila* sequence was aligned using the Clustal W program in BioEdit (version 7.0.5.3; Informer Technologies, Inc., California, USA). The neighbor-joining method performed phylogenetic analysis using MEGA X software (v. 11.0.13)^25^.

### **In vitro bactericidal effect of PAA and H**_**2**_**O**_**2**_**against*A. hydrophila***

#### Test conditions

The bactericidal quantitative suspension test was adapted from BS EN 1276:2009. The test was conducted at room temperature (23–25 ºC) with 300 mg/L water hardness and a 5% organic load. Hard water was prepared by adding 972 mL of distilled water to 12 mL of Solution A (containing 19.84 g of anhydrous MgCl_2_/L and 46.24 g of anhydrous CaCl_2_/L) and 16 mL of Solution B (containing 35.02 g of NaHCO_3_/L). A 5% yeast extract solution was prepared by dissolving 50 g of yeast extract powder in 50 mL of distilled water. Then, 1 mL of this stock solution was added to the test suspension.

#### Test suspension and test (N and N0)

An *A. hydrophila* test (N) was conducted by adjusting the concentration of a subculture of the organism to 1.5–5 × 10^8^ CFU/mL using the 0.5 McFarland standard and a solution of 0.1% tryptone and 0.85% NaCl in distilled water. One milliliter of sterile 5% yeast extract was combined with 8 mL of fresh, diluted disinfectants and mixed thoroughly. The mixture was maintained at 20 °C ± 1 °C for 15 min in a water bath. Afterward, 1 mL of *A. hydrophila* suspension was added, mixed, and left for 5, 30, or 60 min of contact time. At the end of the contact time, 1 mL aliquots were taken and mixed with 8 mL of neutralization solution (containing 3.0 g/L lecithin, 30.0 g/L Tween 80, 30.0 g/L saponin, 5.0 g/L sodium thiosulfate, 1.0 g/L histidine, 1.0 g/L tryptone, and 8.5 g/L NaCl), followed by the addition of 1 mL of sterile distilled water. The mixture was mixed well and left at 20 °C. After a 5-minute neutralization period, 1.0 mL of the neutralization solution was serially diluted to 10^8^. Duplicate 1 mL aliquots of each dilution were plated on tryptic soy agar and incubated at 37 °C for 48 h. The microbial effect (ME) of the tested disinfectants was calculated by subtracting the log of the viable count (CFU) after disinfectant action (Na) from the log of the initial count in the test suspension (before exposure to disinfectants) (N0). A disinfectant was considered legitimate microbicidal action if the viable bacterial count was reduced by 5 logarithms (R).

### In vivo experimental set-up

#### Ethical approval

The experimental procedures and all methods followed the relevant guidelines and regulations established by the Research Ethics Committee of the Faculty of Veterinary Medicine, Cairo University, Egypt (Vet CU 25122023842). This study is reported following ARRIVE guidelines.

#### Experimental design

**Fig. 1:**
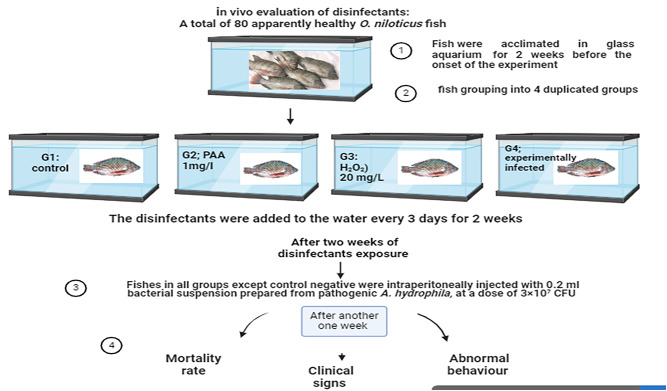
Scheme of the experimental setup.

A total of 80 apparently healthy Nile tilapia fish, each weighing approximately 40 ± 10 g, were obtained from a private fish farm in El-Sharqia governorate, Egypt. The fish were transported in oxygen-supplied, water-filled plastic containers to the Department of Veterinary Hygiene and Animal Management, Faculty of Veterinary Medicine, Cairo University. They were acclimated in glass aquaria (30 × 50 × 100 cm) for 2 weeks before the experiment began. The aquaria were equipped with aeration and a thermostable thermometer set to 28 ºC. Before the experimental period, the fish were divided into four groups in duplicates, each with 10 fish, as shown in Fig. 1. The first group (G1) served as the negative control group (without disinfectant application or bacterial infection); the second group (G2) was exposed to PAA at a concentration of 1 mg/L^15^; the third group (G3) was exposed to H_2_O_2_ at a concentration of 20 mg/L^19^; and the fourth group (G4) was the positive control group, infected with *A. hydrophila*. The overall exposure period was three weeks. The fish were fed twice daily with a basal diet containing 30% protein. Water was exchanged, and disinfectants were added every 3 days until the end of the experiment. Fish behavior and mortality were observed and recorded after disinfectant exposure in the G2 and G3 groups. Fish in groups G2, G3, and G4 were challenged with pathogenic *A. hydrophila* after only 2 weeks of the experimental onset. Water samples were collected from each tank after disinfectant application and at the end of the experiment for physicochemical and microbiological examination.

### Post-exposure bacterial challenge

Following 2 weeks of exposure, fish in groups G2, G3, and G4 (the positive control) were injected intraperitoneally with 0.2 ml of pathogenic *A. hydrophila* bacterial suspension at 3 × 10^7^ CFU^26^. All experimentally infected groups were monitored daily for one-week post-injection to observe any abnormal external lesions and mortalities. Following Koch’s postulates, freshly dead fish were subjected to thorough bacteriological examination for bacterial reisolation. Fish were euthanized before injection using buffered tricaine methane-sulfonate (MS-222) at a concentration of 50 mg/L^27^ as a bath immersion.

### Estimation of cortisol level and antioxidant biomarkers

Representative fish samples from each group were euthanized using an overdose of buffered MS-222 (250 mg/L)^28^ to collect hepatic tissue samples at various time points during the experimental period: before exposure to PAA and H_2_O_2_, immediately after exposure (0.5 h) and 1 week post-exposure. The samples were stored and frozen at −80 °C. Hepatic homogenates were prepared from the frozen specimens by mincing and homogenizing 1 gram of liver tissue with 5 mL of a buffering solution (10 mM Tris, pH = 7.4, 0.25 M sucrose, and 1 mM EDTA), and the suspension was centrifuged at 224 g for 10 min. The resulting supernatant was collected for measuring cortisol levels and antioxidant enzyme activity (CAT, SOD, GPx, TAC, and MDA). According to the manufacturer’s protocol, cortisol levels were detected using an enzyme-linked immunosorbent assay (ELISA) kit (Neogen, USA). Catalase activity (CAT) was estimated as described by Clairborne^29^, and total superoxide dismutase (SOD) activity was determined by inhibiting epinephrine autoxidation, as reported by Magwere^30^. Glutathione peroxidase (GPx) activity was measured according to Moin’s method^31^. Total antioxidant capacity (TAC) was assessed as described by Koracevic et al.^32^. Lipid peroxidation levels were assayed by determining the amount of 2-thiobarbituric acid reactive substrates (TBARS), following the method of Kamyshnikov^33^, and expressed as malondialdehyde (MDA) levels. All antioxidant biomarkers were measured using a colorimetric spectrophotometer (Spectronic 15, Moton Roy Company, USA).

### Sampling and microbiological examination of fish muscular tissue

Representative fish samples were collected from the control negative group and twice from each group, following the first exposure to disinfectant (after 3 h) and at the end of the experiment (after bacterial infection). Ten grams of fish muscle samples were aseptically homogenized with Ringer’s solution at each sampling interval to create serial dilutions following APHA^34^. The total bacterial count (TBC) was determined by spreading 0.1 mL from the diluted tubes onto two sets of plate count agar, followed by incubation for 48 h at 35 °C. Another set of inoculated APC plates was incubated at 7 °C for 7–10 days to count psychrotrophic bacteria^35^. For the enumeration of *Aeromonas* bacteria, double sets of Aeromonas agar base media and Rimler-Shotts (R-S) were used, with counts taken after 48 h at 30 °C^36^.

### Sampling, physicochemical, and microbiological analyses of fish aquaria water

Water samples were collected in labeled plastic bottles at various times for chemical analysis. Physicochemical water parameters were measured on-site using a pH meter, an oxygen meter, and a conductometer (HACH, HQ30, USA). Total hardness was determined using the “EDTA titrimetric method,” as described by APHA^37^. Quantification of nitrate was carried out using the Spectro-Quant^®^ colorimetric test kit (Cod. 1.149442.0001, Merck), and nitrite (4500-NO_2_^−^) and ammonia (4500-NH_3_F) were measured accordingly.

The microbiological analysis of water involved collecting samples in sterile glass bottles from fish culture tanks before and after disinfectant application (30 min post-application), before water exchange (after 3 days), after water exchange, and finally after bacterial infection. Microbiological testing followed APHA^37^ recommendations. The poured plate method was employed to ascertain the total bacterial count (TBC). A sterile glass Petri plate was filled with 1 mL of the serially diluted water samples, each in triplicate. Approximately 15 mL of melted nutritional agar medium was added to each plate, mixed, and allowed to solidify. The plates were incubated at 37 °C for 48 h. After incubation, the number of colonies per plate of the same dilution was counted, and the mean value was determined.

### Statistical analysis

Shapiro-Wilk tests were first used to determine whether the data distribution was normal and evenly distributed. The Hepatic cortisol and antioxidant biomarkers levels data were analyzed statistically using one-way ANOVA with Dunnett’s post-hoc test and Tukey’s test. While the Kruskal-Wallis test was employed to evaluate the microbial density in fish muscular tissue and the water-quality variables, the Mann-Whitney U-test was employed to identify differences between the groups. All data were analyzed on SPSS v19.0 (SPSS Inc., Chicago, IL), with a statistical threshold set at *p* < 0.05 for all tests. The findings were expressed as the mean ± SE. The least significant difference test was used to determine significance. The main effects were considered significant at *p* < 0.05.

## Results

### *A. hydrophila* isolation and identification

#### Phenotypic characterization

Colonies of *A. hydrophila* appeared as pinpoint yellow on *Aeromonas* agar base media and R-S media. Gram staining revealed gram-negative short motile bacilli. Biochemical reactions for oxidase and catalase tests were positive. Analytical profile indexing (API 20E) confirmed *A. hydrophila* isolation with an identity percentage (%id) of 98.4, and the code result was 7,006,127 (Table 1).

**Table 1:** Analytical profile index (API) 20E of isolated *A. hydrophila*.

API 2O E TEST		*A. hydrophila*
Biochemical tests	Reactions/Enzymes	
ONPG	ß-galactosidase	+ve*
ADH	Arginine Di Hydrolase	+ve
LDC	Lysine Decarboxylase	+ve
ODC	Ornithine Decarboxylase	−ve*
CIT	Citrate utilization	−ve
H_2_S	H_2_S production	−ve
URE	Urease	−ve
TDA	Tryptophane Deaminase	−ve
IND	Indole production	−ve
V.P	Acetoin production (Voges Proskauer)	−ve
GEL	Gelatin	+ve
GLU	Fermentation / oxidation Glucose	+ve
MAN	Fermentation / oxidation Mannitol	+ve
INO	Fermentation / oxidation Inositol	−ve
SOR	Fermentation / oxidation Sorbitol	−ve
RHA	Fermentation / oxidation Rhamnose	−ve
SAC	Fermentation / oxidation Saccharose	+ve
MEL	Fermentation / oxidation Melibiose	−ve
AMY	Fermentation / oxidation Amygdalin	+ve
ARA	Fermentation / oxidation Arabinose	+ve
OX	Cytochrome-Oxidase	+ve
NO_2_	NO_2_ production	+ve

* −ve, negative reaction; +ve, positive reaction.

#### Molecular identification and sequencing analyses

**Fig. 2:**
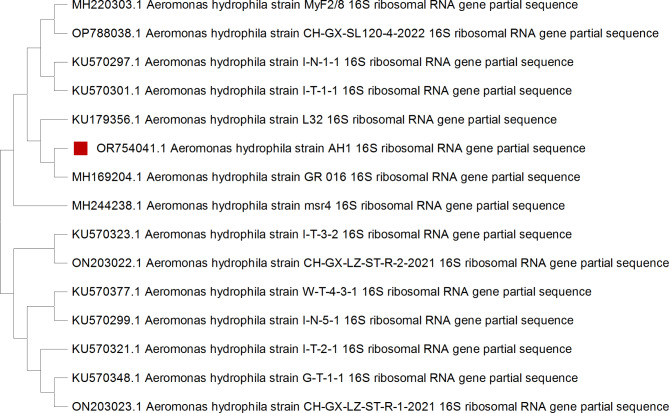
Phylogenetic tree constructed using neighbors-joining method based on the partial *16S rRNA *gene sequence of *A.hydrophila*.

The universal *16 S rRNA* gene confirmed the isolation of *A. hydrophila*. The obtained sequence of the amplified DNA fragment was blasted on NCBI and registered in GenBank with accession number OR754041.1. Sequence alignment and phylogenetic analyses confirmed the identity of the sequenced isolate, as it clustered in the same clade as other *A. hydrophila* strains. These sequences exhibited 98.11% similarity with those isolated from stripped catfish and shrimp in Bangladesh (MH220303.1-MH244238.1), 98.11% similarity with those from *Procambarus clarkii* and estuarine water in India (OP788038.1-MH169204.1), and 97.76% similarity with sequences from crab-cultured water in China (KU570297.1-KU570301.1-KU179356.1) (Fig. 2).

### In vitro bactericidal activity of PAA and H_2_O_2_ against *A.hydrophila*

The results in Table 2 showed that the logarithmic reduction of the average bacterial count of A.*hydrophila* at 1 mg/L PAA achieved a 5-log reduction after 30 min of contact time, while 20 mg/L H_2_O_2_ achieved a 5-log reduction after a contact time of 5 min.

**Table 2:** The mean bactericidal effect (log reduction) of PAA and H2O2 against  *A. hydrophila*.

		Vc1	Vc2	*N* AND N0	
Test-suspension (*N* and No):	10^7^	118	124	*N* = 147.272 × 10^*7*^, *lg.N = 9.16N0 = N/10 = lg N0 = 8.16*	
	10^8^	40	*42*		
*EGY-VIROX* 5% *(PAA* 1 mg / l)					
Contact Time	VC1	VC2	NA Na = 10c/n	lgNa	Lg R (M.E) *(No = 8.16)*
1 min	>300	>300	1380	3.47	4.69
5 min	>300	>300	1380	3.47	4.69
30 min	170	<30	1000	3	5.16
*AQUAPLUS 50% (*H_2_O_2_ 20 mg/l)					
1 min	>300	> 300	1380	3.47	4.69
5 min	200	< 30	1150	3.06	5.1
30 min	130	< 30	1000	2.9	5.26

N: Number of colony-forming units (CFUs) per ml of bacteria in the suspension used in the test. = C/ (n1 + 0.1n2)10^−7^, where: C is the sum of Vc values considered.

n1 is the number of Vc values considered in the lower dilution, i.e. 10^−7^.

n2 is the number of Vc values considered in the higher dilution, i.e. 10^−8^.

N_0_: Number of CFUs per ml in the test mix., N0 = N/10.

Na: The count of the survived bacteria after the treatment of disinfectant = 10c/n.

c is the sum of Vc values considered; and n is the number of Vc values taken into account.

The log reduction (R) = (LgN_0_ - lgNa) must be ≥ 5 to pass the test.

VC: Number of viable CFU on plate.

### Evaluation of fish sensitivity to PAA and H_2_O_2_

Fish in the PAA group (G2) exhibited momentary abnormal swimming behavior, characterized by rapid movement from one side of the aquarium to another and an increase in the escape reflex immediately after adding PAA. These signs rapidly disappeared within 10 min of exposure, and the fish returned to their normal state. The H_2_O_2_-exposed fish group (G3) did not show any abnormalities in movement or behavior. No mortalities were recorded in either the PAA or H_2_O_2_ exposed groups following exposure.

### Post-exposure challenge and bacterial reisolation

**Fig. 3:**
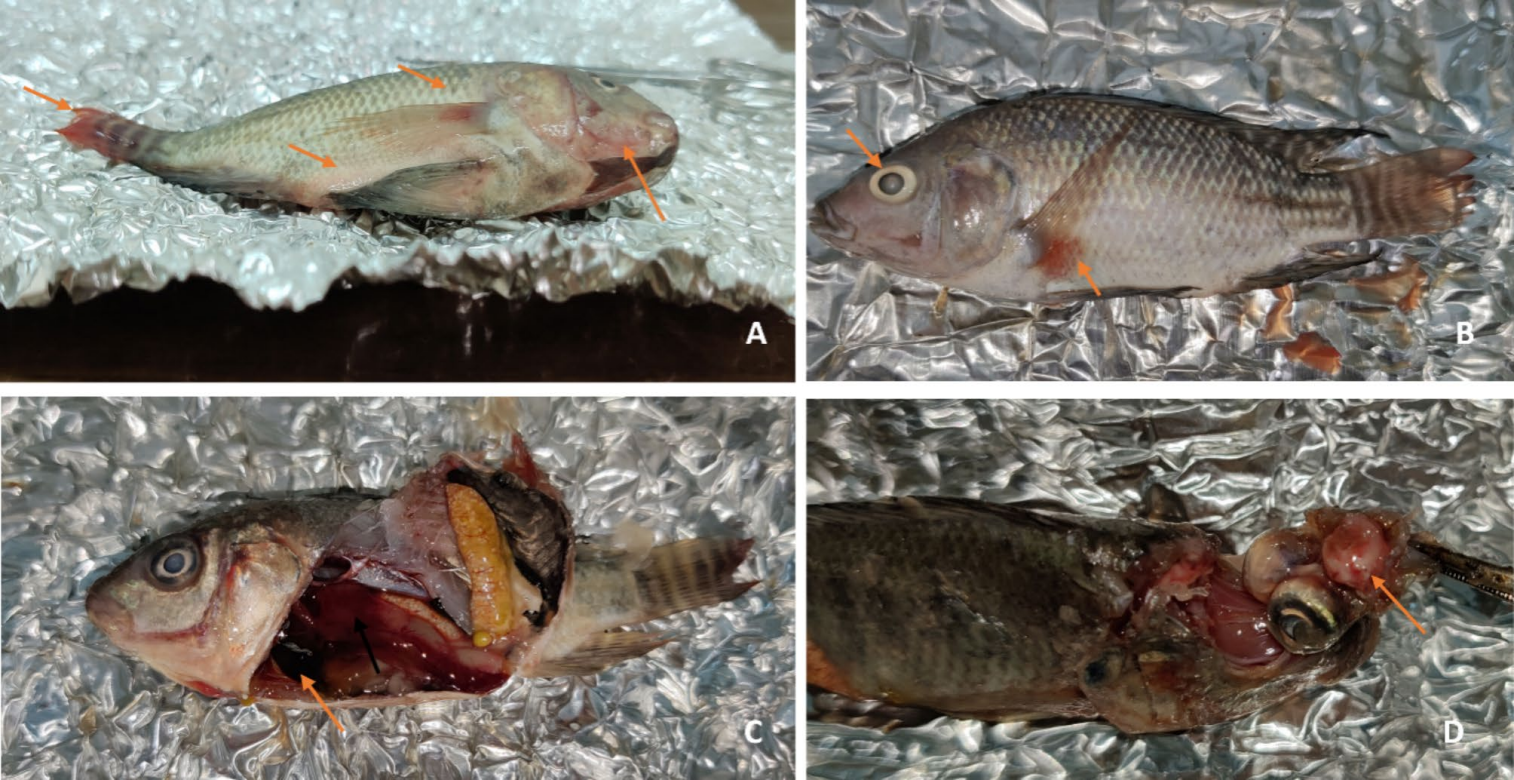
Control positive experimentally infected fish in fourth group (G4) showed typical signs of *A. hydrophila* infection in the form of extensive petechiae on base of fins, isthmus and belly with eroded caudal fin (**A, B**), hemorrhages and congestion in internal organs and brain (**C, D**).

The protective effect of the disinfectants was evaluated by mortality percentage and clinical signs observed in experimentally infected fish in groups G2 and G3. In the PAA-exposed group (G2), injected fish exhibited 20% mortality, with physical changes including gasping and abnormal swimming behavior but no evident external lesions except for darkening. In contrast, the H_2_O_2_-exposed group (G3) showed no mortalities, abnormal behaviors, or clinical signs. Typical *A.* *hydrophila* was re-isolated and identified from the control positive fish group (G4), which displayed skin darkening, detached scales, extensive petechiae on the base of fins, isthmus, and belly, eroded caudal fins, and eye cataracts. Postmortem examination revealed hemorrhagic and congested gills, liver, kidney, brain, and gall bladder, with mortalities reaching up to 90% (Fig. 3).

### Hepatic cortisol levels

**Fig. 4:**
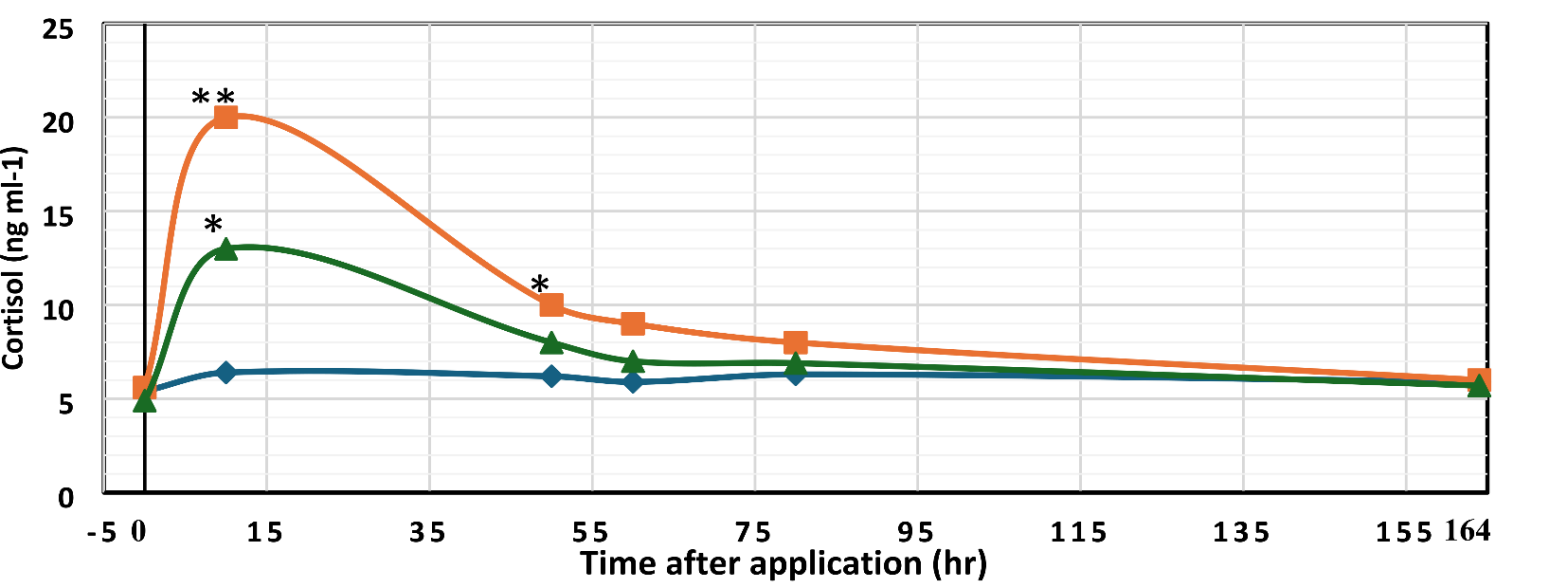
Mean hepatic cortisol levels (n = 3) from control group (blue diamond), PAA exposed group (red square) and H_2_O_2_ exposed group (triangle). The measurements performed before the exposure, after exposure and one week after exposure: An asterisk (*) & (**) denotes significant differences between the control and the exposed groups (*P* < 0.05). Values expressed as mean SE via one-way ANOVA with Dunnett’s post-hoc test.

Compared to the control group, both PAA and H_2_O_2_ groups exhibited a significant increase in cortisol levels immediately after disinfectant exposure. Notably, PAA exposure resulted in a significantly higher increase in hepatic cortisol levels than H_2_O_2_. Fish displayed aggressive or evasive behavior when PAA was added to the tanks. These increases in cortisol levels declined over time, returning to normal levels by the end of the disinfectant exposure period for both PAA and H_2_O_2_ (Fig. 4).

### Hepatic antioxidant biomarkers levels

**Fig. 5:**
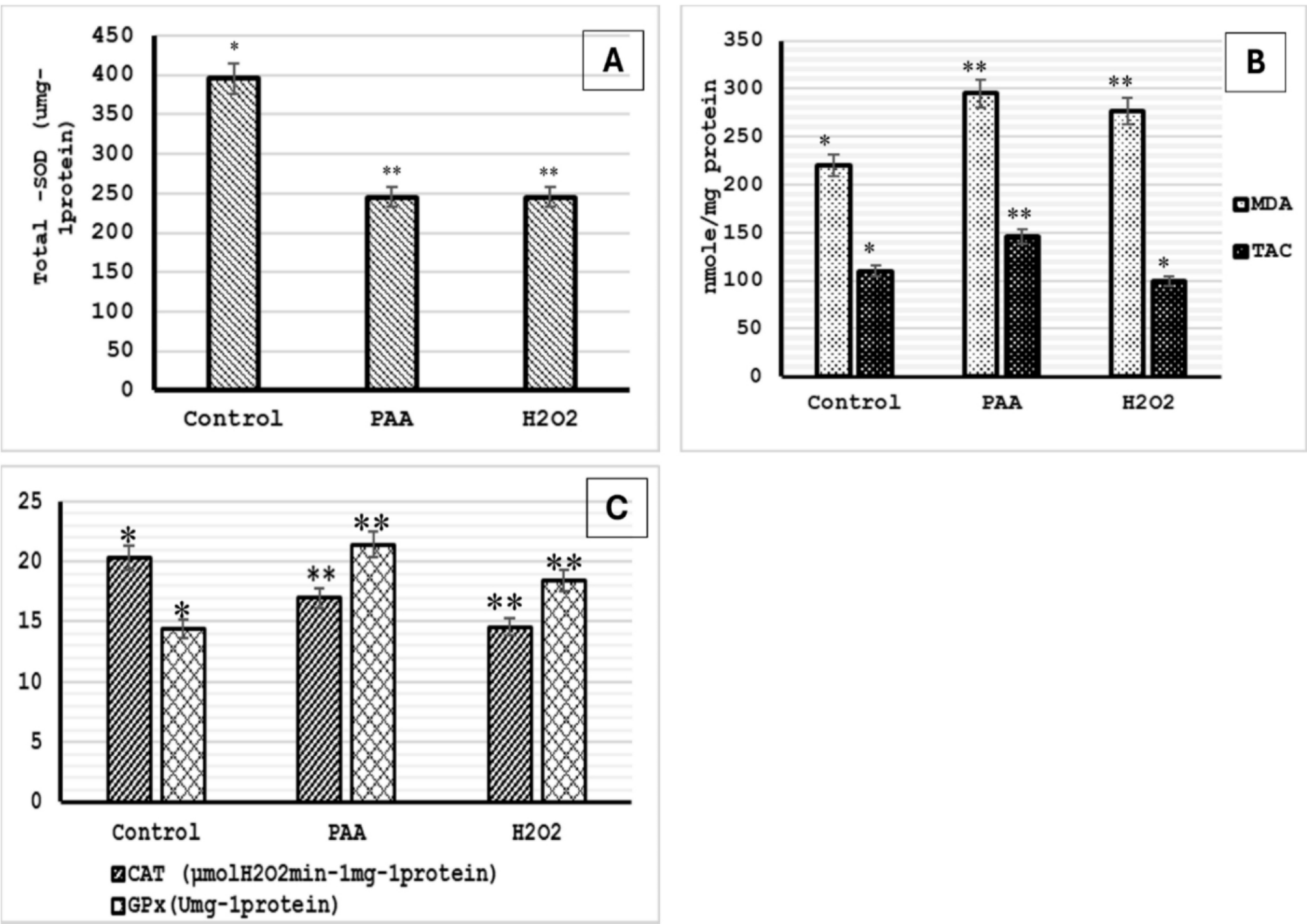
(**A**) Mean hepatic SOD levels, (**B**) Mean hepatic MDA & TAC levels and (**C**) Mean hepatic CAT & GPx levels. The measurements performed after one week of exposure: An asterisk (*) & (**) denotes significant differences between the control, PAA and H_2_O_2_ treated groups (*P* < 0.05. Values expressed as mean SE of three individual fish. one-way ANOVA followed by with Tukey’s test.

In the present study, Fig. 5 represents the hepatic antioxidant biomarkers, which showed a significant decrease in total SOD and CAT enzymatic activity in groups exposed to PAA and H_2_O_2_ compared to the control group. However, GPx enzymatic activity was significantly higher in the PAA and H_2_O_2_ exposed groups than in the control group. Conversely, the MDA level was significantly increased in the PAA and H_2_O_2_ exposed groups compared to the control group. The TAC level showed a significant increase only in the PAA-exposed group compared to both the control and H_2_O_2_ groups.

### Assessment of microbial density in fish muscular tissue

The antimicrobial effects of PAA and H₂O₂ in fish muscles, either before or after infection with *A.hydrophila*, are displayed in Table 3. The results showed that fish groups exposed to PAA significantly reduced TBC (3.07 and 4.17 log_10_ CFU/g), *Aeromonas* spp. (1.17 and 3.28 log_10_ CFU/g), and psychrotrophic bacterial count (2.53 and 3.10 log_10_ CFU/g), either before or after infection, compared to the control and H₂O₂-exposed groups. However, H₂O₂ had a higher inhibitory microbial effect than PAA, either before or after infection. In this regard, fish exposed to H₂O₂ showed the lowest significant mean microbial counts in their muscle samples for total bacterial count (TBC) (2.53 and 3.87 log_10_ CFU/g), *Aeromonas* spp. (1.14 and 2.52 log_10_ CFU/g), and psychrotrophic bacterial count (2.03 and 1.72 log_10_ CFU/g), compared to control samples (4.63, 1.41, and 3.63 log_10_ CFU/g), respectively.

**Table 3:** Effect of PAA and H_2_O_2_ on microbial load (log10 CFU/g) in fish muscles before and after infection.

	Bacteriological examination		
Groups	**TBC**	***Aeromonas*spp.**	**Psychrotrophic bacterial count**
**Before infection**			
G1	4.63 ± 0.218^a^	1.41 ± 0.091	3.63 ± 0.218^a^
G2	3.07 ± 0.029^b^	1.17 ± 0.173	2.53 ± 0.259^b^
G3	2.53 ± 0.075^c^	1.14 ± 0.086	2.03 ± 0.017^b^
*P* value	0.027	0.413	0.050
**After infection**			
G1	4.63 ± 0.218	1.41 ± 0.091^c^	3.63 ± 0.218^a^
G2	4.17 ± 0.088	3.28 ± 0.142^a^	3.10 ± 0.041^b^
G3	3.87 ± 0.186	2.52 ± 0.116^b^	1.72 ± 0.141^c^
*P* value	0.094	0.027	0.027

TBC = Total bacterial count; ^a–e^Means with different superscripts within the same column significantly (*p* < 0.05) different using Kruskal Wallis Test. Values represent the mean of 3 independent replicates ± SE. G1: control negative group; G2: PAA group; G3: H_2_O_2_.

### Physicochemical and microbiological examination of fish aquaria water

**Fig. 6:**
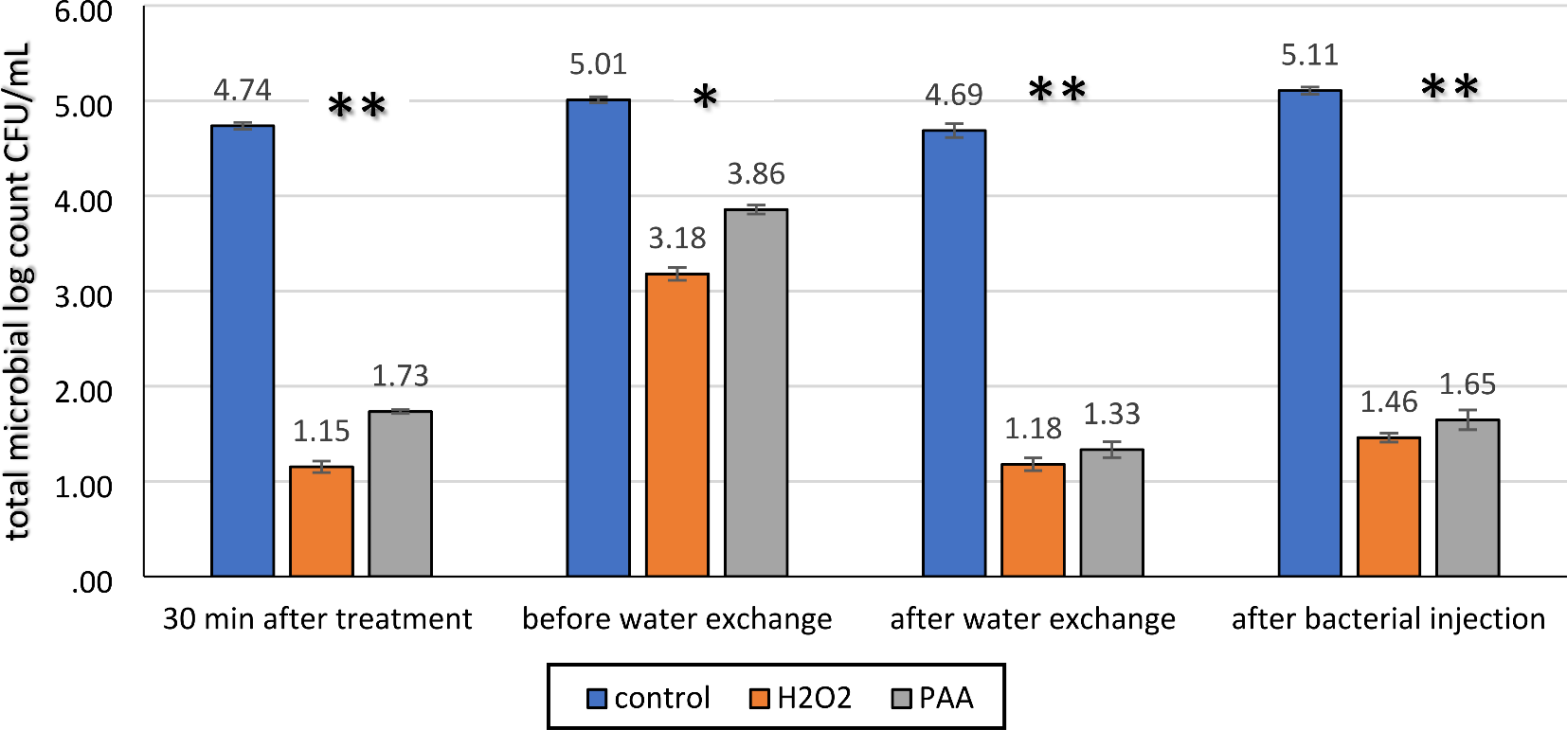
Total microbial count (CFU ml^−1^) measured in water samples from: control, H_2_O_2_, and PAA groups at different sampling time: 30 min after treatment, 72 h. before water exchange, 72 h. after water exchange, and last sampling after bacterial injection. Bars represent mean values ± SE, each GP including technical replicates. An asterisk (*) & (**) denotes significant differences between the control and the treated groups (*P* < 0.05, *p* < 0.001, respectively).

The physicochemical analyses of the collected water samples showed a decrease in pH and an increase in dissolved oxygen concentration after the application of PAA and H₂O₂ disinfectants in the second (G2) and third (G3) groups, respectively, compared to the control group (Table 4). The microbiological analyses assessed the total bacterial count in fish culture water as CFUs before and after disinfectant application and following the bacterial challenge. A significant difference (*p* < 0.05) was observed between the control group and the PAA and H₂O₂ groups at different sampling times: after disinfectant application (30 min), before and after water exchange, and after bacterial infection. The control untreated water samples had a total aerobic bacterial density up to 4–5 times higher, which decreased by nearly 76% in H₂O₂-treated water samples and by 64% in PAA-treated water samples. The total microbial count did not increase significantly after the bacterial challenge in the PAA and H₂O₂-treated groups, as shown in Fig. 6.

## Discussion

Most aquaculture systems use permanent or intermittent water disinfection to manage or remove pathogenic organisms, enhancing fish growth conditions and performance. These water hygiene techniques range from the periodic use of chemical agents to continuous disinfection by UV radiation and ozone production^19^. Chemical disinfectants are predominant synthetic agents employed for water disinfection and disease prevention. Numerous disinfectants are used in aquaculture, particularly for cleaning facilities and equipment and maintaining hygienic conditions during the production cycle^38^. Their application frequency varies from seven days to several months, as reported by other studies^7,8,39^. For our investigation, we utilized PAA and H_2_O_2_ in fish aquaria at prophylactic concentrations of 1 mg/L and 20 mg/L, respectively, twice per week.

Recently, PAA has been used as a practical, eco-friendly, and cost-effective chemical for disinfection and oxygenation. It acts as a broad temperature-tolerant alternative antibacterial agent in freshwater aquaculture systems against a wide range of mycotic, parasitic, and bacterial fish diseases, including *Saprolegnia parasitica*, *Ichthyophthirius multifiliis*, and *Flavobacterium columnare*^12,15,40,41^. Moreover, H₂O₂ has proven its efficacy as a broad-spectrum antimicrobial agent against gram-negative bacteria, parasites, fungi, and viruses^42,43^. In addition to its role as a disinfectant, H₂O₂’s capacity for quick oxygenation could be vital, especially when oxygen levels decrease in response to temperature changes^44^. Furthermore, it has a longer half-life than other reactive oxygen species, serving as a cost-effective oxygen source on aquaculture farms. Few references mentioned the use of H₂O₂ in both freshwater and saltwater for bioremediation^45,46^.

The in vitro study of antimicrobial activity can be highly beneficial in estimating the most effective antibacterial agents for protecting and treating fish in aquatic environments^47^. The bactericidal analyses of water samples conducted in this study, as shown in Table 2, revealed that a concentration of 1 mg/L of PAA against *A. hydrophila* resulted in a 5-log reduction after 30 min, as reported by other authors^13,16,41^. These studies showed that 1 ppm PAA inhibited the in vitro growth of pathogenic bacteria, including *Aeromonas salmonicida*, *Lactococcus garvieae*, and *Yersinia ruckeri*. Nevertheless, exposure to 20 mg/L of H_2_O_2_ reduced the number of living *A. hydrophila* cells by five logs after only 5 min of contact. This indicates that both PAA and peroxides demonstrated significant bacterial log reduction^42,48^. Our results contrast with those of Meinelt et al.^49^, who reported that PAA achieved more bacterial cell reduction than H_2_O_2_.

Waterborne chemicals such as PAA and H_2_O_2_ may act as chemical stressors, causing some behavioral and systemic physiological changes as a strong response to counteract this stress. In our study, fish in the PAA-exposed group (G2) exhibited irregular swimming patterns that rapidly disappeared once conditioning occurred^17^. The H_2_O_2_-exposed fish (G3) did not show any abnormal behavior, which contrasts with the findings of Avendaño-Herrera^50^, where the H_2_O_2_-exposed fish group showed dramatic changes in behavioral and respiratory patterns, which quickly returned to normal. No mortalities were recorded in the PAA and H_2_O_2_-exposed groups, confirming their wide safety margin^48,51^.

*A. hydrophila* isolate was recovered from a previous tilapia mass kill for the experimental challenge. It was identified biochemically using analytical profile indexing (API 20E) with an identity percentage (%id) of 98.4 and genotypically by *16 S rRNA* gene sequencing analysis, which confirmed its classification in the family *Aeromonadaceae* as *A. hydrophila*, with accession number OR754041.1 (Table 1; Fig. 2). PAA and H_2_O_2_ disinfectants have proven effective in controlling bacterial diseases such as Aeromoniasis (*A. hydrophila*), Vibriosis (*V. harveyi* and *Vibrio alginolyticus*), and Photobacteriosis (*Photobacterium damselae*), with high survival rates in various fish species, including Nile tilapia (*Oreochromis niloticus*), Rainbow trout (*Oncorhynchus mykiss*), Atlantic salmon (*Salmo salar*), and Gilthead seabream (*Sparus aurata*)^17,52,53^. In our study, the antibacterial effect of PAA and H_2_O_2_ was confirmed and tested in vivo through a post-exposure challenge experiment. The results revealed that fish injected in the PAA group (G2) experienced a 20% mortality rate with skin darkening and some behavioral alterations, which are often preliminary responses to harmful stimuli^54^. On the other hand, no mortalities or abnormal behaviors were observed in the fish injected in the H_2_O_2_ group (G3), contrasting with Avendaño-Herrera et al.^50^, who reported cumulative mortalities up to 100% post-challenge with *Tenacibaculum maritimum* in the H_2_O_2_-exposed group (30–240 mg/L for 30 min). These differences in mortality rates may be attributed to variations in disinfectant concentrations, exposure duration, infection severity, fish species, and culturing conditions. The control positive group (G4), experimentally infected without disinfectant exposure, showed typical external clinical and postmortem signs of *A. hydrophila*, as observed in studies by Abdel-Moneam et al.^5^ and Ayoub et al.^54^.

Cortisol is a reliable indicator of stress in different fish species^15,55^. In the present investigation, the first exposure to PAA elicited visual behavioral responses indicative of stress, reflected in elevated cortisol levels. These results align with Soleng et al.^56^, who reported moderate stress responses and a rise in cortisol levels after exposure to 1 ppm PAA for 5 min under different husbandry conditions. Similarly, there was a significant rise in cortisol levels after exposure to H_2_O_2_, consistent with observations in Atlantic salmon^57,58^. However, cortisol levels steadily declined over time, and basal cortisol levels were restored in both groups (G2, G3) 24 h after exposure, indicating a non-severe reaction to the disinfectants. Continuous exposure to PAA or H_2_O_2_ causes fish to adapt and downregulate their stress response, making it negligible^59^. This confirms our findings that fish in the PAA (G2) and H_2_O_2_ (G3) groups, when exposed to bacterial challenges and receiving sustained periodic treatments, showed noticeable improvements in overall health. Our results agree with earlier research on exposure to peroxides^18,19^ and PAA^13–16^.

Oxidative stress primarily occurs due to fish exposure to environmental stressors, resulting from decreased antioxidant levels or increased reactive oxygen species (ROS), which can cause pathological conditions^60,61^. Antioxidant enzymes and other redox molecules scavenge excess ROS and suppress cellular oxidative damage, and any changes in antioxidant enzymatic activity act as distress biomarkers^62,63^. Oxidative stress can be assessed by measuring levels of lipid peroxidation, protein oxidation/nitration, and DNA/RNA damage through biomarkers such as MDA, SOD, and CAT. Malondialdehyde (MDA) is the most mutagenic product of polyunsaturated fatty acid peroxidation, leading to lipid degradation in cell membranes, cell damage, and cell death^64^. Superoxide dismutases (SODs) and catalase (CAT) are widely used as stress markers in aquatic environments. They form the front line of defense, protecting animal cells from ROS-mediated damage. SODs remove peroxides and lipid hydroperoxides by catalyzing superoxide radicals (O_2_^−^) into molecular oxygen and H_2_O_2_, reducing excess O_2_^−^ that can destroy cells^65^. CAT then splits H_2_O_2_ into water and oxygen^66^.

PAA has been categorized as a powerful oxidizing agent and a significant source of ROS, reflected in antioxidants’ enzymatic activity. PAA-based disinfectants can induce a transitory state of oxidative stress in exposed fish before fully degrading their components^13^. Compared to the control, the recorded simultaneous increase in hepatic MDA activity in the PAA- and H_2_O_2_-exposed groups indicated the accumulation of ROS, with consequent lipid damage. This could contribute to the decrease in the catalytic activity of SOD and CAT and the accumulation of free radicals, which increase ROS generation by tissues and enhance lipid peroxidation due to harmful products^67^. Glutathione peroxidase (GPx) is a second highly potent antioxidant enzyme that reverses oxidative damage by hydrolyzing H_2_O_2_ into reduced glutathione^68^. Total antioxidant capacity (TAC) is used to evaluate the overall non-enzymatic defense mechanism against ROS.

The enhancing effect of PAA on TAC and GPx levels results from the decline in redox balance due to oxidative stress. It is suggested that antioxidants are mobilized to neutralize the changes induced by the moderate stress effects of PAA exposure and protect the fish by maintaining homeostasis^56,69^. This upregulation in GPx may compensate for the decrease in SOD and CAT enzymatic activity. The same result was reported by Soleng et al.^56^.

On the other hand, in this study, there was a decrease in SOD, CAT, and TAC, with a significant increase in MDA levels in the H_2_O_2_-exposed group. H_2_O_2_ induces hepatic oxidative stress injury in fish due to forming hydroxyl radicals via Fenton-like reactions, contributing to lipid peroxidation and a shortage in antioxidant defense mechanisms, especially at higher doses^70^. The elevation of GPx in the H_2_O_2_-exposed group could be an attempt by hepatocytes to counteract the adverse oxidizing effects of peroxides on hepatic tissue. Similar observations have been reported by Wang et al.^71^. Despite the temporary changes in antioxidant biomarker levels in fish exposed to PAA and H_2_O_2_, these changes were reasonable and acceptable and did not result in significant mortality or permanent abnormal behavior.

*Aeromonas* spp. are emerging as major human pathogens with significant public health implications^72^. Aeromoniasis in humans can cause symptoms ranging from mild to dysentery-like diarrhea, as well as meningitis and septicemia^5^. It mostly occurs after consuming improperly cooked seafood or drinking contaminated water^73^. Therefore, using chemical disinfectants with broad protection margins in aquaculture systems is essential to prevent disease entry or spread without harming the fish or posing a public health risk.

The antibacterial effects of PAA and H_2_O_2_ on fish muscles are displayed in Table 3. The results showed that the PAA-exposed group and H_2_O_2_ exposed group experienced a significant reduction in TBC, and psychrotrophic bacterial counts, before infection, while after infection there is a significant reduction in TBC, *Aeromonas* spp., and Psychotropics bacterial count, compared to the control group. This is consistent with Liu et al.^13^, who reported that PAA (1 mg/L) reduced the total aerobic bacterial count in water tanks by approximately 90%, positively impacting fish tissue. The findings agree with Chi et al.^8^, who found that *Pangasius hypophthalmus* fish exposed to PAA at different concentrations (10, 220, 50, and 120 ppm) for 10, 20, and 240 s achieved a high reduction rate in *E. coli* muscle count (0–1.0 log CFU/g), with a minimal decrease in lactic acid bacteria. These results may be attributed to the high efficacy of PAA against Gram-negative bacteria compared to Gram-positive bacteria^74^ and the negative impact of organic matter in fish-culturing water on the disinfectant efficacy of PAA.

In this study, the H_2_O_2_-exposed groups demonstrated the maximum microbial inhibitory effect, both before and after the bacterial challenge. These results are consistent with El-Dosoky and Mostafa^75^, who found that treatment of chicken meat with H_2_O_2_ (1% and 2%) for two minutes significantly decreased TBC, coliforms, and *S. aureus*. Furthermore, H_2_O_2_ significantly reduced bacterial count below the detectable limit and extended the shelf life of seabass fillets during chilling storage for 25 days. Additionally, Pedersen and Pedersen^38^ reported that all isolated bacteria were 100% sensitive to H_2_O_2_.

Regarding the physicochemical water analyses, PAA- and H_2_O_2_-exposed groups exhibited significantly lower pH values than the control group shortly after disinfectant application. The pH decrease can be attributed to PAA-based products stabilized by an acidified mixture of H_2_O_2_ and acetic acid, resulting in a low-pH solution. Liu et al.^14^ previously reported that PAA causes a pH reduction, which may directly influence PAA toxicity in zebrafish embryos. In this study, PAA therapy caused a temporary pH drop, with daily pH variations of ≤ 0.2 in all groups. Dissolved oxygen concentrations were highest in the PAA and H_2_O_2_-exposed groups, while the control group had the lowest. H_2_O_2_ acts as an efficient disinfectant even at lower concentrations. According to Bogner et al.^19^, doses of 15.8 mg/L H_2_O_2_ consistently raised oxygen levels in the tank water from approximately 50% to above 100% saturation after four hours. The water quality in the current study was within the EPA’s recommended limits^76^.

Our findings (Table 4) showed that, regardless of the sampling period, both the PAA and H_2_O_2_ treatment groups and the control group exhibited comparable levels of ammonia concentration. In contrast, nitrate and nitrite levels decreased in the treated groups compared to the control. In line with these results, Liu et al.^13^ demonstrated that water quality in flow-through aquaculture systems improved when PAA-based disinfectants were used twice weekly at 1 mg/L, compared to continuous use at 0.2 mg/L, without impairing fish performance.

**Table 4:** Physicochemical water quality parameters in control, PAA, and H_2_O_2_ groups before and after application (mean ± SE).

Parameters	Control	PAA	H_2_O_2_	*p*-value
PH	7.68 ± 0.05^a^	7.35 ± 0.04^b^	7.48 ± 0.04^b^	0.039*
Dissolved Oxygen (DO) (mg/L)	7.90 ± 0.03^c^	9.31 ± 0.11^a^	8.74 ± 0.06^b^	0.027*
Ammonia (NH_4_) (mg L^−1^)	0.11 ± 0.01	0.12 ± 0.00	0.12 ± 0.01	0.370
Nitrite (NO_2_)(mg L^−1^)	0.19 ± 0.05^a^	0.15 ± 0.03^c^	0.17 ± 0.03^b^	0.027*
Nitrate (NO_3_) (mg L^−1^)	1.49 ± 0.02 ^a^	1.38 ± 0.10^b^	1.37 ± 0.09^b^	0.063
Total hardness	693 ± 3.51^b^	709 ± 4.93^ab^	715 ± 2.98^a^	0.069
Conductivity (MS/cm)	43 ± 1.6^b^	54 ± 1.45^a^	55.3 ± 2.02^a^	0.063

^a–e^Means with different superscripts within the same row. SE: standard error. *The results are significant (*p* < 0.05) using Kruskal Wallis Test.

Disinfectants eliminate bacteria in fish aquaria and alter the composition and functioning of microbial communities after disinfection. As shown in Fig. 6, PAA and H_2_O_2_ application reduced microbial diversity in the water^50,51^. Microbial counts in the PAA group were higher than in samples treated with H_2_O_2_. Throughout the treatments, H_2_O_2_-exposed water samples showed a substantial decrease of around 76% in microbial density, while PAA-exposed samples exhibited a 64% reduction. This contrasts with Liu et al.^13^, who reported a 90% reduction in total aerobic bacterial count with PAA in water tanks. Untreated water samples showed a significant rise in total aerobic bacterial density, increasing by 4–5 times. There was a statistically significant difference (*p* < 0.05) between the control and treated disinfectant groups (PAA and H_2_O_2_) at all sampling times: 30 min after treatment, 72 h before water exchange, 72 h after water exchange, and after bacterial injection. After the bacterial challenge, a non-significant increase in total microbial count was observed, indicating the protective efficacy of PAA and H_2_O_2_ during bacterial infection.

## Conclusion

In summary, the present investigation demonstrated that using PAA at a concentration of 1 mg/L and H_2_O_2_ at 20 mg/L, applied twice weekly, offers effective disinfection by significantly reducing suspended and overall aerobic bacterial density in fish culture water without causing drastic changes in physicochemical parameters. Additionally, it decreased the total microbial load in fish muscles and reduced mortalities in fish challenged with *A. hydrophila*, thereby improving fish health and performance over time. PAA- and H_2_O_2_-exposed groups showed a temporary increase in cortisol levels and alterations in antioxidant enzymatic activity, either through an increase (TAC, GPx, and MDA) or decrease (SOD and CAT), with no recorded mortalities. This suggests that exposure to PAA and H_2_O_2_ may induce minimal stress. However, further analysis is required to assess these disinfectants’ long-term safety and impact on fish health.

## Electronic supplementary material

Below is the link to the electronic supplementary material.

Supplementary Material 1

## Data Availability

The data supporting this study’s findings are available from the corresponding author upon request. for the data that is deposited in GenBank: weblink of the accession number (OR754041.1) https://www.ncbi.nlm.nih.gov/nucleotide/OR754041.1?report=genbank&log$=nuclalign&blast_rank=1&RID=8WZ311 × 0013.
